# Noninvasive classification of physiological and pathological high frequency oscillations in children

**DOI:** 10.1093/braincomms/fcaf170

**Published:** 2025-05-02

**Authors:** Lorenzo Fabbri, Eleonora Tamilia, Margherita A G Matarrese, Linh Tran, Saleem I Malik, Dave Shahani, Cynthia G Keator, Steven M Stufflebeam, Phillip L Pearl, M Scott Perry, Christos Papadelis

**Affiliations:** Neuroscience Research, Jane and John Justin Institute for Mind Health, Cook Children’s Health Care System, Fort Worth, TX 76104, USA; Department of Bioengineering, University of Texas at Arlington, Arlington, TX 76019, USA; Division of Newborn Medicine, Department of Pediatrics, Boston Children’s Hospital, Harvard Medical School, Boston, MA 02115, USA; Division of Epilepsy and Clinical Neurophysiology, Department of Neurology, Boston Children’s Hospital, Harvard Medical School, Boston, MA 02115, USA; Neuroscience Research, Jane and John Justin Institute for Mind Health, Cook Children’s Health Care System, Fort Worth, TX 76104, USA; Department of Bioengineering, University of Texas at Arlington, Arlington, TX 76019, USA; Research Unit of Intelligent Health Technology for Health and Wellbeing, Department of Engineering, Università Campus Bio-Medico di Roma, Rome 00128, Italy; Neuroscience Research, Jane and John Justin Institute for Mind Health, Cook Children’s Health Care System, Fort Worth, TX 76104, USA; Neuroscience Research, Jane and John Justin Institute for Mind Health, Cook Children’s Health Care System, Fort Worth, TX 76104, USA; Neuroscience Research, Jane and John Justin Institute for Mind Health, Cook Children’s Health Care System, Fort Worth, TX 76104, USA; Neuroscience Research, Jane and John Justin Institute for Mind Health, Cook Children’s Health Care System, Fort Worth, TX 76104, USA; Athinoula A. Martinos Center for Biomedical Imaging, Massachusetts General Hospital & Harvard Medical School, Charlestown, MA 02129, USA; Division of Epilepsy and Clinical Neurophysiology, Department of Neurology, Boston Children’s Hospital, Harvard Medical School, Boston, MA 02115, USA; Neuroscience Research, Jane and John Justin Institute for Mind Health, Cook Children’s Health Care System, Fort Worth, TX 76104, USA; Neuroscience Research, Jane and John Justin Institute for Mind Health, Cook Children’s Health Care System, Fort Worth, TX 76104, USA; Department of Bioengineering, University of Texas at Arlington, Arlington, TX 76019, USA; Burnett School of Medicine, Texas Christian University, Fort Worth, TX 76129, USA

**Keywords:** electrical source imaging, high-density electroencephalography, high frequency oscillations, magnetoencephalography, drug-resistant epilepsy

## Abstract

High frequency oscillations have been extensively investigated as interictal biomarkers of epilepsy. Yet, their value is largely debated due to the presence of physiological oscillations, which complicate distinguishing between normal versus abnormal events. So far, this debate has been addressed using intracranial EEG data from patients with drug-resistant epilepsy. Yet, this approach suffers from inability to record control data from healthy subjects and lack of whole brain coverage. Here, we aim to differentiate physiological from pathological high frequency oscillations using non-invasive whole brain electrophysiological recordings from children with drug-resistant epilepsy and typically developing controls. We recorded high-density EEG and magnetoencephalography data from 47 controls (median age: 11 years; 25 females) and 54 children with drug-resistant epilepsy (median age: 14 years, 33 females). We detected high frequency oscillations (in ripple frequency band) semi-automatically and localized their cortical generators through electric or magnetic source imaging. From each ripple, we extracted a set of temporal, morphological, spectral and spatial features. We then compared the features between ripples recorded from the epileptic brain (further distinguished into those from epileptogenic and non-epileptogenic regions) and those recorded from the control group (normal brain). We used these features to cross-validate a Naïve-Bayes algorithm for classifying each ripple recorded from children with epilepsy as coming from an epileptogenic region or not. We observed more high frequency oscillations on EEG than magnetoencephalography recordings (*P* < 0.001) both in the epilepsy and control groups. Physiological high frequency oscillations (recorded from controls) showed lower power, shorter duration and less variability (in both amplitude and duration) than those recorded from the epilepsy group (*P* < 0.001). Inter-channel latency of physiological ripples was longer compared to ripples from the epileptogenic regions (*P* < 0.01), while it was similar to the ripples from non-epileptogenic regions (*P* > 0.05). Ripples from epileptogenic regions showed larger extent than those from non-epileptogenic regions or from the control group (*P* < 0.001). The classification model showed an accuracy of 73%, with negative and positive predictive values of 73% and 70% (*P* < 0.0001), respectively, in classifying high frequency oscillations from the drug-resistant epilepsy group (as either epileptogenic or not). Our study indicates that physiological high frequency oscillations, recorded from the healthy brain, have distinct temporal, morphological, spectral and spatial features compared to those generated by the epileptic brain. The differentiation of pathological from physiological high frequency oscillations through non-invasive full-head techniques may augment the presurgical evaluation process of children with drug-resistant epilepsy and lead to better postsurgical seizure outcomes.

## Teaser

Fabbri *et al*. have studied high frequency oscillations in children with drug-resistant epilepsy and healthy controls using non-invasive methods. They found that high frequency oscillations from the healthy group were shorter, less powerful, more stable and generated by smaller areas than those from people with epilepsy.

## Graphical Abstract

**Graphical Abstract:**
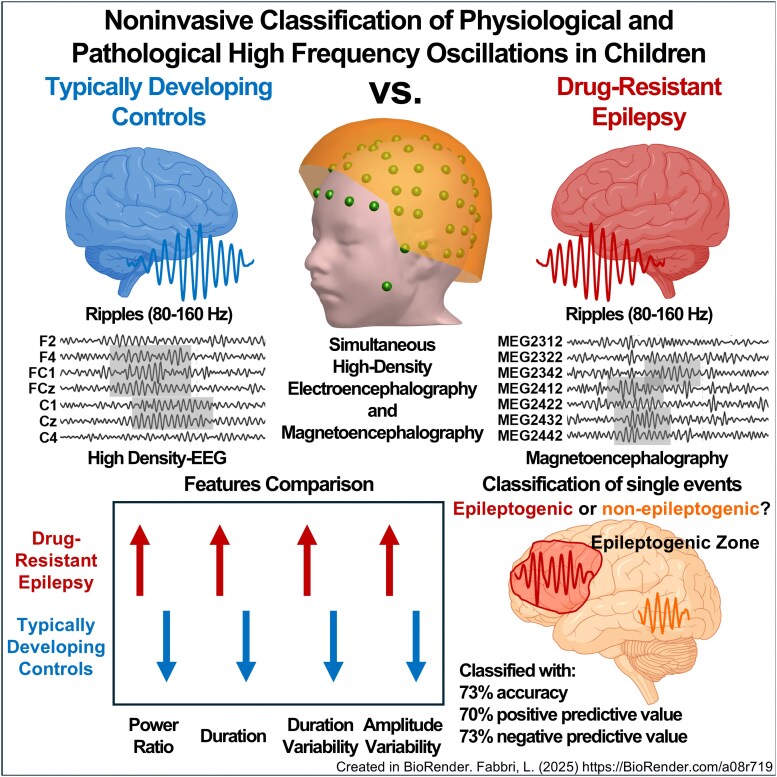

## Introduction

High frequency oscillations (HFOs) have been extensively studied as interictal biomarkers of epilepsy.^1-8^ Yet, their presurgical value in localizing the epileptogenic zone (EZ) is still largely debated since the cortical areas that generate HFOs often extend beyond the actual EZ.^9-14^ This is due to the presence of physiological HFOs produced by normal (non-epileptogenic) brain regions. Such a physiological contamination occurs predominantly in the ripple frequency band (80–250 Hz)^9,11,14,15^ at rest. Physiological HFOs are also linked to specific cognitive processes, such as memory retrieval and consolidation, or they are evoked by tasks or stimuli.^9,14,16-22^ Discriminating physiological from pathological ripples is central to their use as presurgical interictal biomarkers of epilepsy. Several attempts^9,16,18,20-23^ have been made over the years to investigate this purpose; however, no strategy currently exists to differentiate these two entities unambiguously. Two major approaches have been undertaken to identify pathological HFOs. One is to create a normative HFO atlas in the normal (non-epileptogenic) brain that accounts for the physiological distribution of HFOs when looking at the epileptic brain.^24,25^ This method has been proposed for both children^24,25^ and adults.^10,13^ Through this approach, research groups have shown that the presence of physiological ripples on intracranial EEG (iEEG) varies substantially between brain regions, with the highest rates to occur in the occipital, sensorimotor and mesiotemporal regions.^10,13^ Another approach is to extract a variety of signal properties (i.e. amplitude, duration, spread and spectral frequency) and use them to recognize fingerprints of pathological or physiological HFOs. However, the findings reported in the literature to date have not demonstrated consistency; while some studies indicate distinct group differences—mainly in terms of amplitude, duration and/or frequency^21,26^—between presumed pathological and physiological ripples, other research has suggested that these two categories exhibit overlapping characteristics, leading to difficulty in distinguishing them.^27^ Consequently, despite previous attempts to clarify this issue, current evidence indicates that a reliable method for differentiating pathological from physiological ripples remains elusive.

The distinction between physiological and pathological HFOs has been primarily addressed so far by using iEEG data from patients with drug-resistant epilepsy (DRE), which inherently suffer from two main limitations: (i) the inability to record control data from healthy subjects (without epilepsy or other neurological disorders) to characterize physiological HFOs; and (ii) the lack of whole brain coverage, as iEEG electrodes are planned to cover the brain regions that are considered potentially epileptogenic or pathological, and thus infrequently record from truly healthy brain tissue. Given the lack of healthy control data, previous studies considered physiological HFOs as those recorded from brain areas without any recorded epileptiform activity or structural abnormality.^10,28,29^ This definition is limited by the fact that epileptogenic areas can also generate physiological HFOs: the presence of both types of HFOs in specific cortical regions (e.g. sensorimotor, hippocampus and associative cortices) complicates the differentiation.^30^ A prior iEEG study differentiated physiological and epileptogenic HFOs based on their timing relative to speech stimuli, and showed that surgical damage to areas exhibiting physiological HFOs was linked to postsurgical speech impairment.^31^ Interestingly, when task-induced HFOs were studied, it was shown that cognitive tasks did not modulate the rate of pathological HFOs.^32^

Moreover, when physiological HFOs were defined on iEEG using data from patients without epilepsy or task-induced data from patients with DRE, differences within pathological HFOs were observed in amplitude, duration and frequency. However, the small sample size^21^ or significant overlap between groups^27^ limits the generalizability of the findings. The possibility to record HFOs with scalp EEG and/or magnetoencephalography (MEG) has boosted HFO research and broadened their applicability over the past decade. Several groups^33-36^ have demonstrated that, in patients with DRE, HFOs can be detected and localized non-invasively using scalp EEG and/or MEG data. The minimal risk involved with these modalities paves the way for characterizing physiological HFOs in the healthy brain. In addition, scalp EEG and MEG provide a full head coverage allowing a complete spatial characterization of HFOs in both patients with epilepsy and healthy subjects. One retrospective study^37^ investigated ripples on scalp EEG data from children with and without epilepsy showing that amplitude and frequency differed between ripple co-occurring with physiological rather than pathological sharp transients (i.e. vertex wave versus interictal epileptiform discharge, IED). However, there is a complete lack of prospective scalp EEG or MEG studies on healthy subjects for the characterization of physiological HFOs and their distinction from pathological HFOs recorded from patients with DRE.

This study aims to differentiate physiological from pathological HFOs non-invasively by comparing data from children with DRE with typically developing children (TDC). For this purpose, we collected high-density EEG (HD-EEG), MEG and MRI data from a DRE and a TDC cohort, aiming to: (i) generate cortical distribution maps of physiological HFOs (ripples) in the normal human brain of TDC children; and (ii) identify differences in the properties of HFOs generated by the healthy and the epilepsy brain.

## Materials and methods

This is a prospective, non-interventional, multi-centre study involving Cook Children's Medical Center (CCMC) (Fort Worth, TX, USA) and Boston Children's Hospital (BCH) (Boston, MA, USA). IRB protocol was approved by North Texas Regional IRB. Consent forms were obtained from all participants. All participants were recruited between June 2020 and November 2023.

### TDC children

TDC were recruited at CCMC. Inclusion criteria were: (i) age over six months and under 19 years; and (ii) ability to understand the study's procedures and comply with them. Exclusion criteria were lack of sleep data, pregnancy, diagnosis of any genetic syndrome, ferromagnetic materials in the body, chronic pain, head injury, history of seizures, brain tumour, cerebrovascular accident and brain surgery.

### Children with DRE

Children with DRE were recruited at CCMC and BCH. Inclusion criteria were: (i) age over six months and under 19 years; (ii) DRE diagnosis; (iii) candidates for surgical resection with iEEG extra-operative monitoring (previous brain surgery is acceptable); and (iv) ability to understand and comply with the study's procedures. Exclusion criteria were lack of sleep data, pregnancy, diagnosis of any genetic syndrome and ferromagnetic materials in their body.

### HD-EEG and MEG recordings

#### Data collection

##### CCMC

MEG data were recorded with a 306 sensors MEG system (102 magnetometers, 204 planar axial gradiometers; Elekta Neuromag, Helsinki) inside a single-layer magnetic shielded room (MSR). HD-EEG data were recorded with a Geodesic Sensor Net EEG system (128 or 256 channels). Recordings were performed simultaneously except if otherwise specified. More details about the protocol are provided elsewhere.^38^

##### BCH

MEG and HD-EEG data from BCH patients were recorded as part of their clinical care at Athinoula Martinos Center for Biomedical Imaging (AMC; Charlestown, MA, USA). Recordings were performed in a three-layer MSR with a 306-channel MEG system (VectorView, Elekta Neuromag, Finland). HD-EEG was recorded with 70-channel electrode caps (EASYCAP Brain Products, Germany) plus two electrodes covering temporal regions (T1/T2). More details about the protocol are provided elsewhere.^36,38-40^

At both sites, data were recorded for 10–12 sessions (4-min each; ≥1000 Hz sampling rate). Data from TDC were recorded only at CCMC.

T1-weighted structural MRI sequences from all participants were acquired using a Siemens Skyra 3T scanner (three-dimensional Magnetization Prepared Rapid Acquisition Gradient Echo or MPRAGE sequences). Standard co-registration procedures were followed for MEG and HD-EEG.^38^

### Ripple detection

We selected segments of non-rapid-eye-movement (NREM) sleep for analysis given the increase in HFOs and minimal presence of motion artefacts during this stage.^41-44^ Ripple detection was performed, separately for HD-EEG and MEG data, via automated detection followed by human visual review for rejection of false positives and artefacts.^35^ HD-EEG data were reviewed in average montage. For MEG, given the high-frequency noise present on magnetometers, ripple analysis was performed only on signals recorded with gradiometers (204 channels).

Data quality was evaluated using standard (1–70 Hz, 10 s/page) and HFO display settings (80–160 Hz, 4 s/page). Segments containing artefacts, high-frequency noise or technical disruptions were visually inspected (L.F.), marked and excluded from further analysis. Frequencies above 160 Hz were excluded to ensure the highest signal-to-noise ratio,^35^ and to set a cut-off frequency more than three times below our minimum sampling rate.

**Figure 1:**
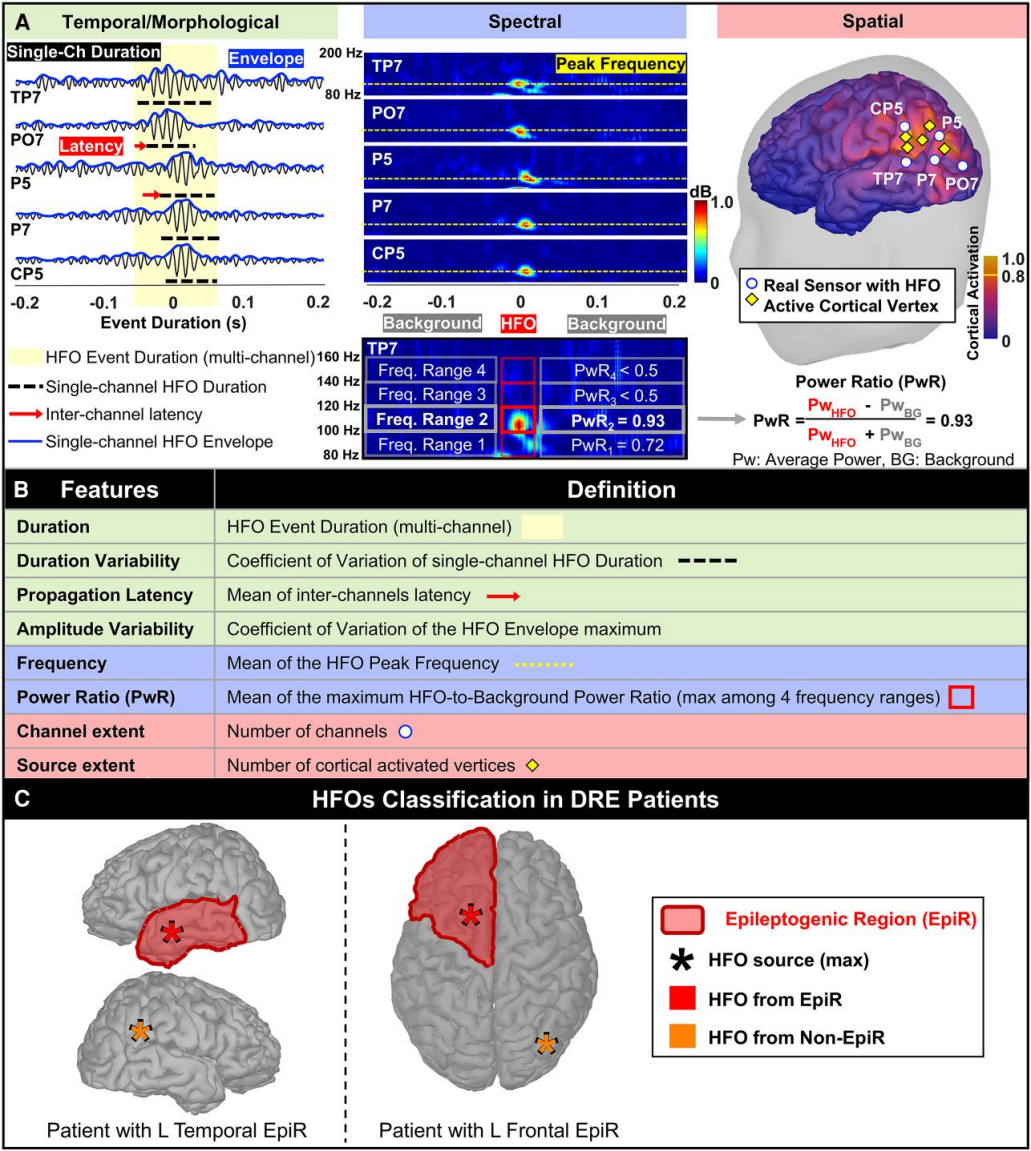
**HFO characterization through temporal/morphological, spectral and spatial features**. (**A**) Each panel (from left to right) show: (i) EEG signals filtered between 80 and 160 Hz on an expanded timescale of 400 ms (ripple time window in yellow); (ii) time–frequency analysis showing the ripple ‘island’ (isolated spectral peak) based on the power ratio computation; and (iii) source localization, on a subject-specific cortical surface, of the HFO, showing the active real sensors (channels) and cortical sources (vertices). (**B**) Comprehensive list of features and their descriptions. Temporal/morphological (green), spectral (blue) and spatial (red). (**C**) Classification of HFOs from DRE patients. The asterisk represents the maximum value of the wMEM map located in the epileptogenic (red) or non-epileptogenic (orange) region. Ch, channel; EpiR, epileptogenic region; Freq, frequency; HFO, high frequency oscillation; L, left; non-EpiR, non-epileptogenic region.

For HFO detection, we used an in-house algorithm^39^ which we adapted to ensure high sensitivity on HD-EEG/MEG as in previous works.^35,36^ Data were band-pass filtered between 80 and 160 Hz^35^ and fed to the algorithm that detected an HFO when: (i) the envelope's *Z*-score was higher than 3; and (ii) there were at least four oscillations lasting more than 25 ms. We defined an ‘event of interest’ (EOI) when there were at least two (for HD-EEG) or three (for MEG) detections with overlapping duration in different channels. We excluded EOIs detected in 75% (or more) of the channels.^35,39,45^ as likely to be muscle and/or movement artefacts. We then performed time–frequency analysis (TFA; Morlet transform) on all channels during the EOI and identified the channels with a spectrally isolated component in the ripple frequency band (80–250 Hz), which we will call ‘TF-island’. To detect the TF-island (see Fig. 1A), we computed the power ratio defined as the normalized difference between the ripple power and the surrounding background in four 20-Hz frequency bins (between 80 and 160 Hz). A TF-island was detected when the power ratio was above 0.5 in one frequency bin. For each EOI then, we defined as HFO channels those identified through the envelope analysis plus those with the TF-island. False positive detections were subsequently eliminated through visual review of each EOI by two reviewers (L.F. and E.T.) both in time and time–frequency domains. Careful attention was paid to exclude artefacts following previous guidelines.^46-50^ We minimized human bias in HFO analysis by using an objective, standardized detection pipeline and a blinded review process, where two independent experts reached a consensus on valid events. We regarded as ripples the EOIs that clearly stood out from the surrounding background showing an isolated peak in the time–frequency plane or at least four oscillations in the filtered signal. Artefacts were defined as events that showed very irregular morphology, excessively high amplitude compared to background activity, or high amplitude/frequency variability.^47,49,51^ False positives were identified as signals originating from the cortex that did not resemble HFOs. These included sharp transients, distortions caused by filtering and physiological activities related to sleep. Further analysis was performed only on the visually confirmed HFOs. We will use the term HFO to refer to the multi-channel event as shown in Fig. 1A.

### Source localization

We extracted individual cortical surfaces from the MRIs via CAT12^52^ and constructed realistic head models using OpenMEEG^53^ (three-layer boundary elementary model).^54^ Source space was constrained to the cortex, where signal-to-noise ratio and sensitivity are maximum for HD-EEG/MEG.^55^

Electric and magnetic source imaging (ESI, MSI) were performed independently for HD-EEG and MEG data on the band-pass filtered data (80–160 HZ) using the wavelet Maximum Entropy on the Mean (wMEM),^56^ which is publicly available in Brainstorm^57^ and allows to localize specific scales of interest (i.e. frequency-bands). Data were down sampled to 640 Hz to ensure that the second scale corresponded to ripples (80–160 Hz).^51,58^ Source localization was performed across each HFO time-window. Diagonal noise covariance matrix was estimated from a 250–500 ms artefact-free window before or after the HFO (excluding windows with other HFOs). We obtained a wMEM map for each HFO, where activation values were associated to each cortical vertex and time point. wMEM maps were averaged across time and normalized in amplitude, so that the most active vertex had a value equal to one.^51,58^ The highest amplitude vertices (i.e. normalized amplitude > 0.8) delineated each HFO-source. Finally, we identified the maximum of each wMEM map to define each HFO's anatomical location based on the Desikan-Killiany atlas, obtained through automated cortical parcellation in 68 sub-lobes and six regions (i.e. frontal, central, temporal, parietal, occipital and cingulate). Rates of HFOs (HFOs/min) per cortical regions were computed for each subject as the number of HFOs divided by the duration of the artefact-free segments.

### HFO feature extraction and labelling

We computed the following properties from each HFO (see Fig. 1B): (i) duration (i.e. duration of the multi-channel HFO event); (ii) duration variability (i.e. coefficient of variation, COV, of the single-channel HFO durations); (iii) propagation latency (i.e. average latency between single-channel HFO within an event); (iv) amplitude variability (i.e. COV of the single-channel HFO amplitudes); (v) frequency (i.e. frequency with the highest power, averaged across all HFO channels); (vi) power ratio (i.e. maximum HFO-to-background ratio among four frequency ranges, averaged across all HFO channels); (vii) channel extent (i.e. number of HFO channels); and (viii) source extent (i.e. number of the HFO-source cortical vertices).

HFOs from patients with focal DRE were labelled as generated by epileptogenic (EpiR) or non-epileptogenic regions (non-EpiR) (Fig. 1C). The EpiRs were defined at the lobar level (i.e. frontal, temporal, parietal, central, occipital, or cingulate) based on the following localizing factors, which were determined based on presurgical workup reports: surgically targeted region (i.e. resection, ablation or responsive neurostimulation target), ictal stereotaxic EEG findings, MRI lesion and ictal scalp EEG findings. The lobes pointed out by any of the presurgical tests were considered as part of the EpiR. In patients who had a good postsurgical outcome (see definition below), the EpiR corresponded to the lobe of the resected region. Supplementary Table 1 reports the EpiRs for all included patients and the determinant factors used to define their EpiR (not all factors were available for all patients). Non-EpiRs were defined as the lobes in the opposite hemisphere from the EpiR. HFOs from patients with generalized or diffuse DRE (involving several regions in both hemispheres) were labelled as generated by the ‘Diffuse DRE brain’.

Surgical outcome was assesses based on the patient's most recent follow-up using the Engel classification (classes I–IV)^59^ and categorized into good (Engel I–II) or poor (Engel III–IV) outcome.

### Statistical analysis

We performed Pearson correlation between each HFO feature and the TDC age to assess any developmental trend in the HFO properties over time. Nonparametric one-way analysis of variance (Kruskal–Wallis test) was used to compare HFO rates and features between cortical regions or types of HFOs, followed by a Tukey–Kramer multiple comparison test. Proportions were compared via Chi-square test. Wilcoxon rank-sum was used for unpaired comparisons. MATLAB 2024a was used for statistical analysis. *P*-values below 0.05 were regarded as significant. Results are reported as median (interquartile range: 25th–75th percentile).

#### Classification model

We implemented a supervised classification algorithm to classify individual HFO events. We trained a kernel Naïve-Bayes classifier (10-fold cross-validation) to assess the likelihood of a recorded HFO to belong to a certain class based on our set of features. HFOs recorded from TDC and focal DRE patients were used for cross-validation. HFOs from patients with diffuse or generalized DRE were excluded. Therefore, we had three classes of HFOs: (i) class 1: HFOs from healthy brain (recorded from TDC); (ii) class 2: HFOs from Non-EpiR (recorded from DRE); and (iii) class 3: HFOs from EpiR (recorded from DRE). Since we had multiple classes, we created a receiver operating characteristic (ROC) curve for each class by using a one-versus-all approach, and calculated their area under the curve (AUC). Finally, we assessed the model accuracy to classify the HFOs recorded from the DRE patients as epileptogenic (predicted as class 3) or not (predicted as class 1 or 2). Thus, we considered as: (i) true positive (TP), HFOs from EpiR classified as class 3; (ii) true negative (TN), HFOs from non-EpiR classified as class 1 (healthy) or 2 (non-EpiR); (iii) false positive (FP), HFOs from non-EpiR classified as class 3; and (iv) false negative (FN), HFOs from EpiR classified as class 1 (healthy) or 2 (non-EpiR). Accuracy (ACC) and positive and negative predictive values (PPV, NPV) were computed to evaluate the classification performance. Performance was computed counting each HFO from the DRE group when they were in a validation fold.

## Results

**Figure 2:**
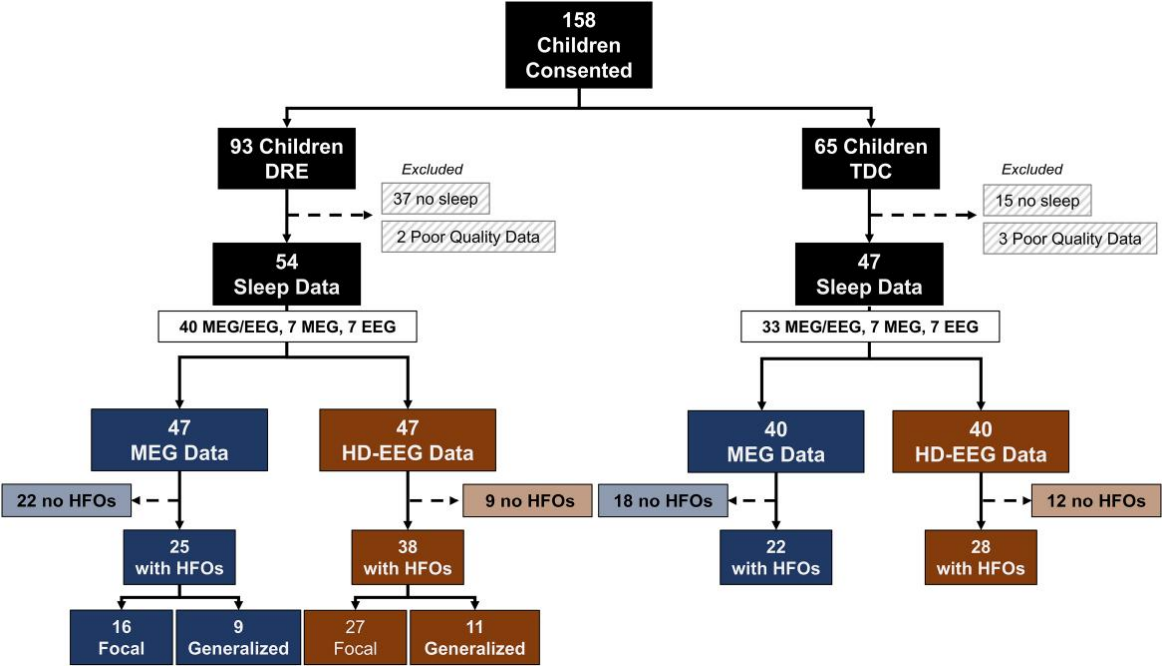
**Flow diagram of participants’ inclusion in the study**. The diagram shows the number of participants recruited for this study for both the DRE and TDC group, the number of sleep recordings acquired with each modality, the number of participants where HFOs were detected and their respective epilepsy classification. DRE, drug-resistant epilepsy; EEG, electroencephalography; HD, high-density; HFO, high frequency oscillation; MEG, magnetoencephalography; TDC, typically developing controls.

We enrolled 65 TDC and 93 children with DRE. We excluded 18 TDC and 39 DRE patients because of high-frequency noise (>80 Hz) on HD-EEG and MEG data or absence of sleep recordings. We thus included 47 TDC [age = 11 (9–15) years; 53% female] and 54 children with DRE (44 from CCMC, 10 BCH) [age = 14 (10–15) years; 61% female]. Figure 2 shows the flow chart of the enrolled and included children, split between TDC and DRE as well as between HD-EEG and MEG. Supplementary Table 4 shows the median duration and interquartile range of the NREM sleep data analysed for each group and recording modality.

### HFO rates in DRE and TDC

**Figure 3:**
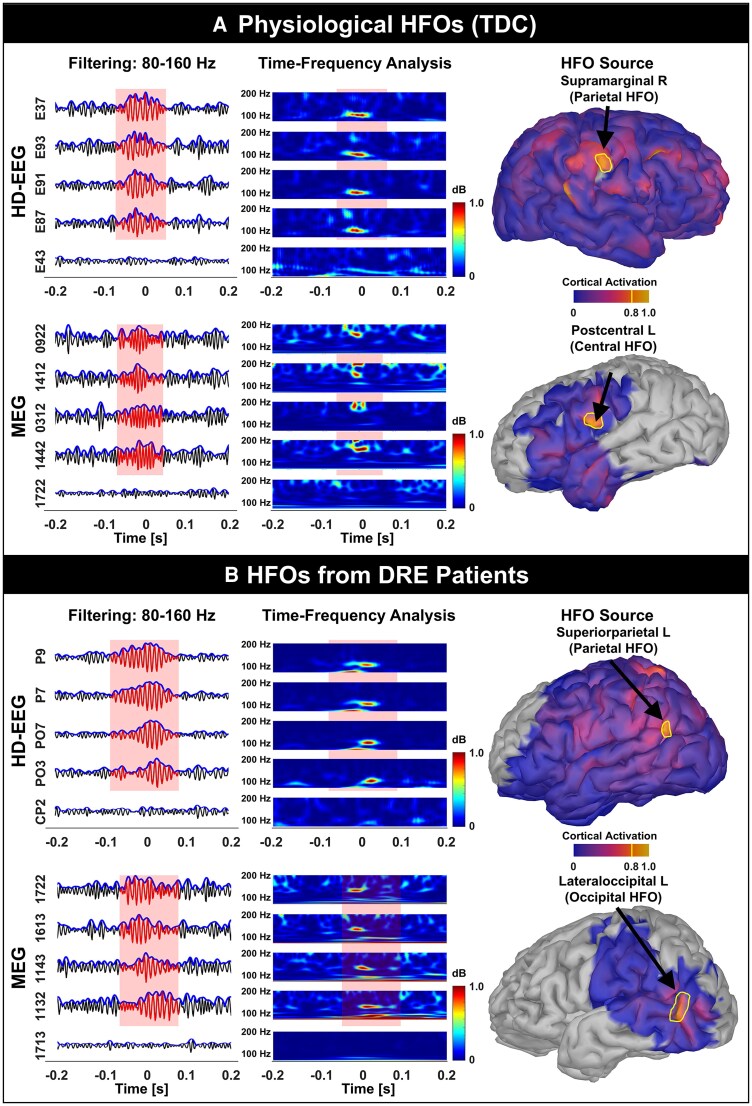
**Examples of HFOs recorded with HD-EEG and MEG**. (**A**) HFOs recorded from two TDC (6- and 12-year-old females). (**B**) HFOs recorded from two children with DRE (Patients 47 and 49 in Supplementary Table 1). Each panel shows one HFO recorded with HD-EEG (*top*) and one with MEG (*bottom*). Within each panel, we report (from left to right): (i) the signals filtered between 80 and 160 Hz on an expanded timescale (ripple time window in red); (ii) time–frequency analysis showing ripple ‘island’ (isolated spectral peak); and (iii) source localization, on the subject's 3D cortical surface, with maximum activation outlined in yellow. DRE, drug-resistant epilepsy; EEG, electroencephalography; HD, high-density; HFO, high frequency oscillation; L, left; MEG, magnetoencephalography; R, right; TDC, typically developing controls.

**Figure 4:**
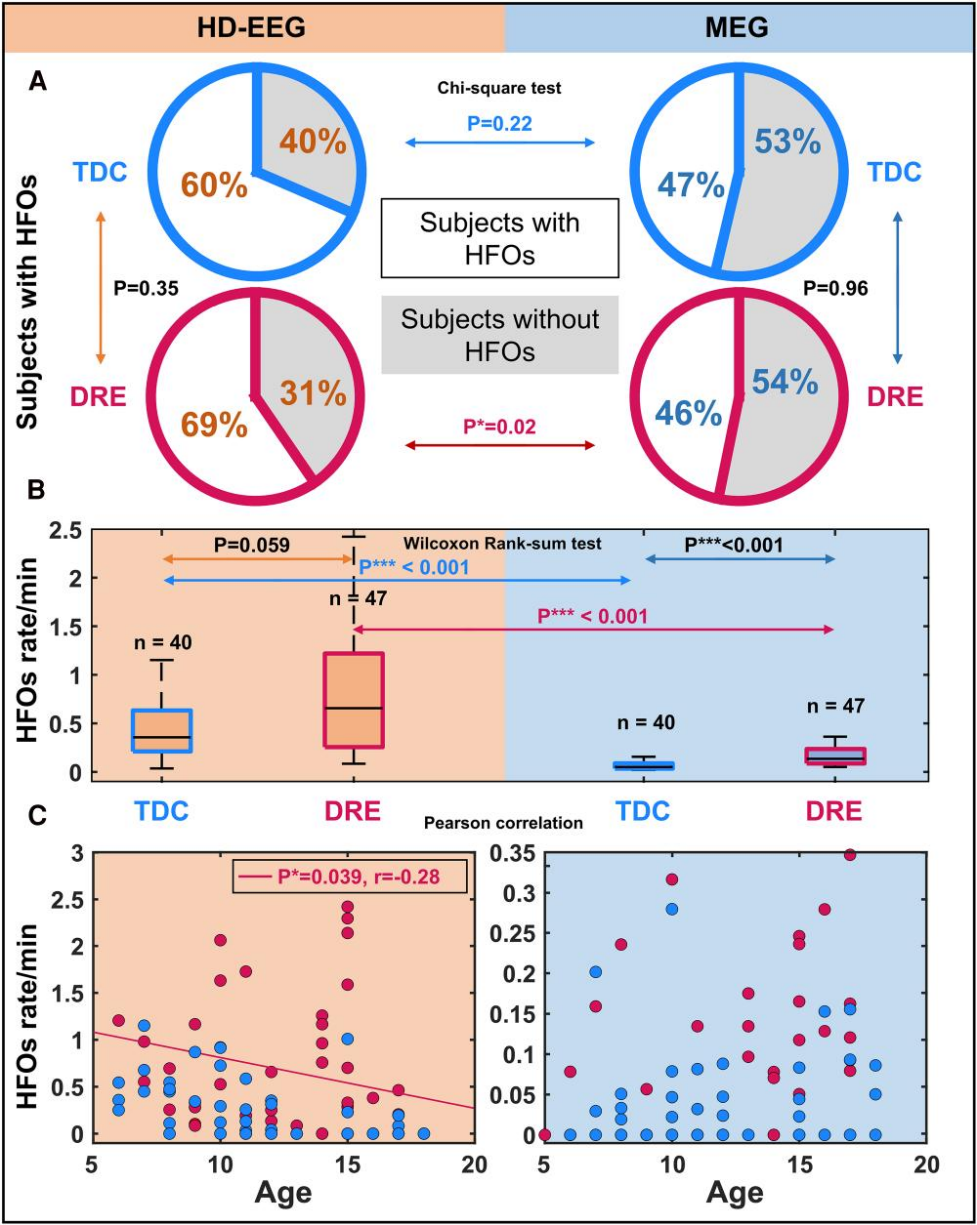
**HFOs on HD-EEG (orange) and MEG (azul) recorded from the TDC (blue) and DRE group (red)**. (**A**) Proportions of subjects presenting HFOs in the different groups are displayed via pie charts. (**B**) Comparisons between HFO rates in TDC and DRE children are displayed via boxplots. (**C**) Correlation of HFO rate with the subjects’ age (Pearson correlation). Regression lines are shown for DRE (red). *r* = correlation coefficient. *P < 0.05; **P < 0.01; ***P < 0.001. DRE, drug-resistant epilepsy; EEG, electroencephalography; HD, high-density; HFO, high frequency oscillation; MEG, magnetoencephalography; TDC, typically developing controls.

In TDC, we found HFOs on HD-EEG in 28 (out of 40) children and on MEG in 22 children. In the DRE group, we found HFOs on HD-EEG in 38 children (out of 47) and on MEG in 25 children. Figure 3 shows examples of physiological HFOs found on HD-EEG or MEG in TDC (Fig. 3A) as well as HFOs recorded from DRE patients via HD-EEG or MEG (Fig. 3B). HFOs were found in similar proportion between TDC and DRE children for both HD-EEG (60% versus 69%; *P* = 0.35) and MEG (47% versus 46%; *P* = 0.96, Fig. 4A). HD-EEG detected HFOs in more DRE patients than MEG (69% versus 46%, *P* = 0.02, Fig. 4A) and with higher rates (0.87 95% CI [0.64–1.1] versus 0.16 95% CI [0.13–0.2] HFOs/min, *P* < 0.001, Fig. 4B). HFO rate was higher in DRE than TDC for MEG (*P* < 0.001, 0.16 versus 0.08 95% CI [0.05–0.11] HFOs/min, Fig. 4B) and showed a similar (nearly significant) trend in HD-EEG (*P* = 0.059, 0.87 versus 0.56 95% CI [0.34–0.9] HFOs/min, Fig. 4B). We observed higher HFO rates in HD-EEG than MEG for both groups (*P* < 0.001, Fig. 4B). No differences were seen between HFO rates in patients with focal versus diffuse or generalized DRE in HD-EEG (*P* = 0.33) or MEG data (*P* = 0.13). For the DRE group, HFO rates correlated (negatively) with patient's age for the HD-EEG (*P* = 0.039, *r* = −0.28) but not for the MEG data (Fig. 4C left). No correlation between HFO rates and patient's age was found in TDC for both HD-EEG and MEG (Fig. 4C right).

### Physiological HFOs (TDC)

The rates of HFOs in the TDC were higher when recorded through HD-EEG than MEG (0.56 versus 0.08 HFOs/min; *P* < 0.001).

#### HD-EEG

**Figure 5:**
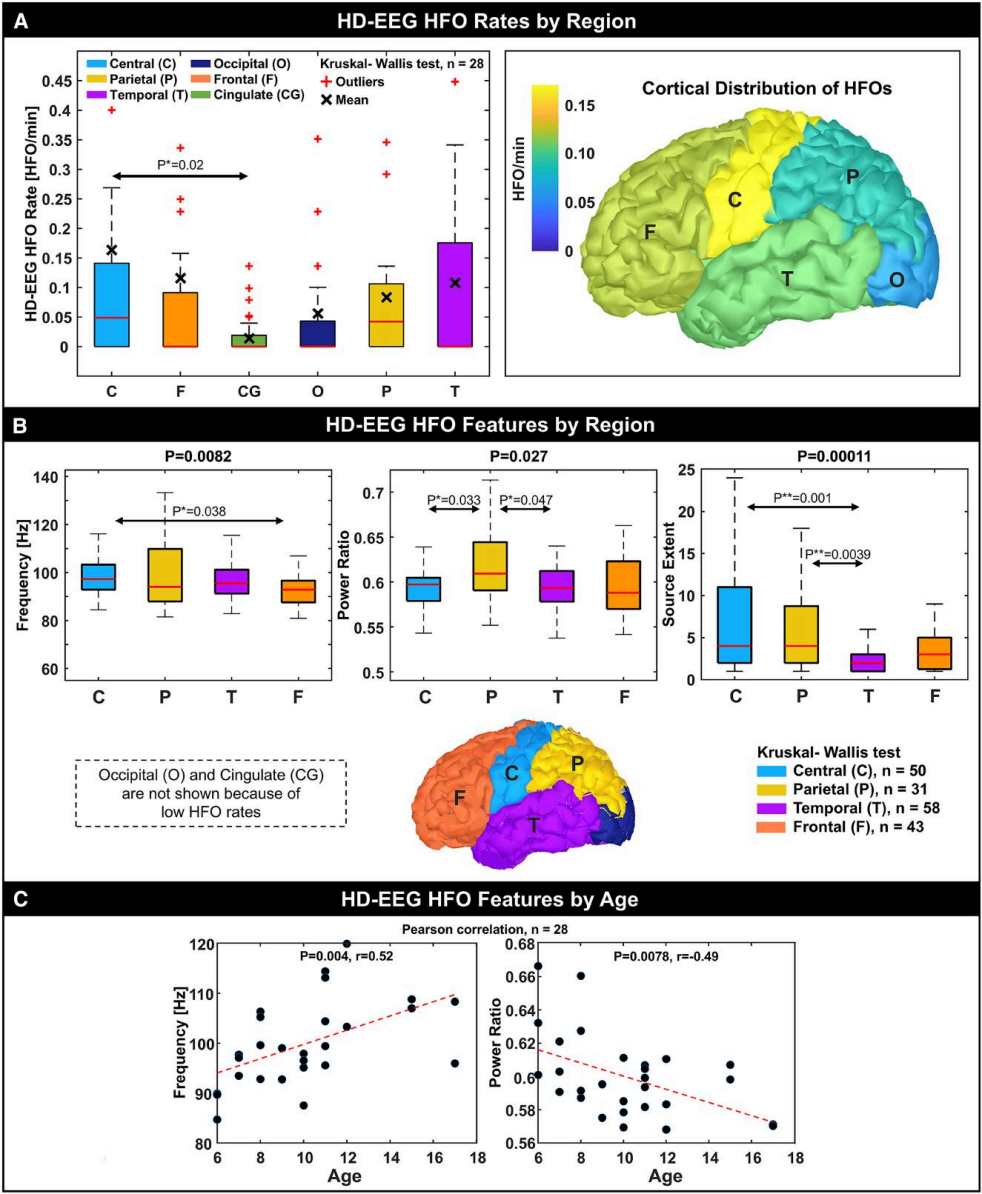
**Physiological HFOs from TDC: HD-EEG findings**. (**A**) Boxplots of HFO rates (HFOs/min) by cortical region and associated distribution projected onto a generic cortical surface colour-coded based on the mean rate per lobe (HFOs/min). (**B**) HFO feature comparisons between regions. Only features showing significant different are shown. Cingulate and occipital regions were excluded due to low HFO rates (median equal to zero). (**C**) Correlation of HFO features with participants’ age. Each data point represents the feature mean between all HFOs recorded for a patient. *r* = correlation coefficient. *P < 0.05; **P < 0.01; ***P < 0.001. EEG, electroencephalography; HD, high-density; HFO, high frequency oscillation.

Figure 5A shows the cortical distribution of HD-EEG physiological HFOs in our TDC cohort demonstrating the highest rates in the central area (pre- and post-central gyrus). When comparing the HFO features between cortical regions (Fig. 5B), we observed: (i) higher frequency in the central HFOs compared to the frontal HFOs (*P* = 0.038); (ii) higher HFO power in the parietal regions than central and temporal regions (*P* = 0.033, *P* = 0.047, respectively); and (iii) larger source extent for the HFOs generated in the central region and parietal regions compared to the temporal (*P* = 0.001, *P* = 0.0039, respectively). Median rates of physiological HFOs in the occipital and cingulate areas were null; thus, these regions were not compared. We found a positive correlation between HFO frequency and the child's age (Fig. 4C; *r* = 0.52, *P* = 0.004), and a negative correlation for the HFO power ratio (Fig. 4C; *r* = −0.49, *P* = 0.0078). No correlation with the age was observed for the other features (*P* > 0.05).

#### MEG

The median rates of MEG HFOs per anatomical region were null in all lobes, except for the temporal, which presented an extremely low mean rate of 0.006 HFOs/min. Thus, inter-regional comparison of HFO characteristics was not performed. A negative correlation was found between the child's age and HFO duration (*r* = −0.49, *P* = 0.021), and no correlation for the other features (*P* > 0.05).

### Comparisons of HFO features in TDC versus DRE

Supplementary Tables 2 and 3 report the results from the comparisons (Kruskal–Wallis) of the HFO characteristics between the four classes of HFOs on HD-EEG and MEG: (i) physiological HFOs generated by the TDC brain (blue); (ii) HFOs generated by non-EpiRs in the DRE brain (orange); (iii) HFOs generated by EpiRs in the DRE brain (red); and (iv) HFOs generated by the diffuse or generalized DRE brain (grey).

### HD-EEG HFOs

**Figure 6:**
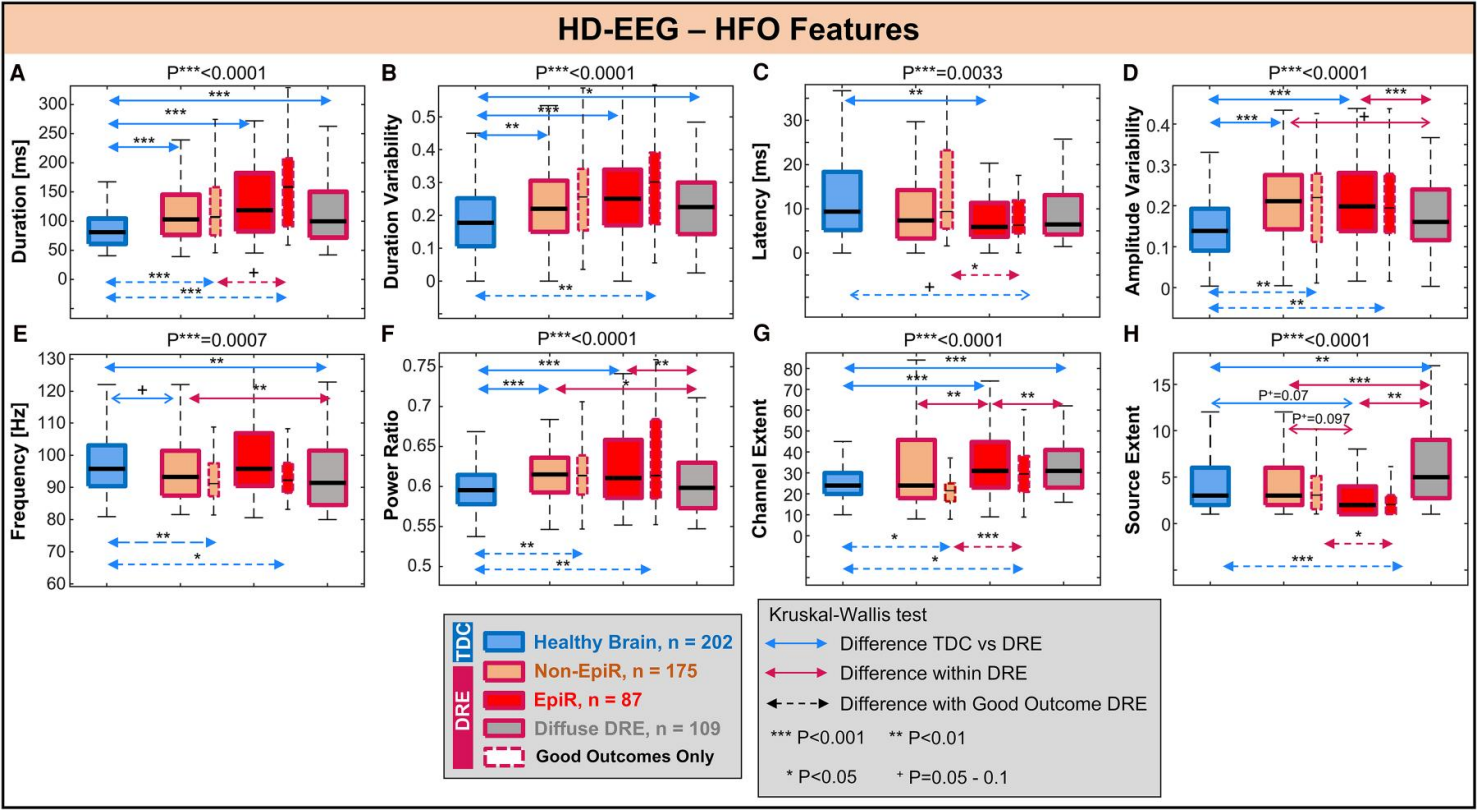
**HD-EEG HFO feature comparison**. (**A–H**) Comparison of each feature between four classes of HFOs: HFOs coming from the healthy brain (blue) of TDC, epileptogenic (EpiR, red) and non-epileptogenic (non-EpiR, orange) regions of DRE patients and patients with diffuse or generalized DRE (diffuse DRE, grey). Dash-line boxplots show results obtained using only DRE patients that had good surgical outcome after surgery (where non-EpiRs are unambiguously defined). DRE, drug-resistant epilepsy; EEG, electroencephalography; EpiR, epileptogenic region; HD, high-density; HFO, high frequency oscillation; non-EpiR, non-epileptogenic region; TDC, typically developing controls.

We found a total of 202 physiological HFOs from TDC with HD-EEG; for the DRE children, we found 87 HFOs from EpiR, 175 from non-EpiR and 109 from the diffuse DRE group. Figure 6 shows the comparison of all the investigated features between the four classes of HFOs on HD-EEG.

#### Temporal and morphological features

Physiological HFOs presented a shorter duration (81 ms) than HFOs from the DRE brain (*P* < 0.001, Fig. 6A, see Supplementary Table 2), whether from EpiR (119 ms), non-EpiR (103 ms) or diffuse DRE brain (100 ms), without differences between DRE HFO classes. When looking at good outcomes only, HFO duration in the EpiR tended to be higher than in non-EpiR (*P* < 0.001). The HFO duration was less variable across channels in physiological HFOs than in HFOs from the DRE brain (*P* < 0.001, Fig. 6B), without differences between the DRE classes. When looking at good outcomes only, the HFO duration variability was lower in physiological HFOs than the HFOs from EpiR (*P* < 0.01), while no differences were seen between the other classes. Physiological HFOs showed a longer propagation latency across channels than HFOs from EpiR (9 versus 6 ms, *P* = 0.003, Fig. 6C), and did not differ from the other HFO classes. HFOs from diffuse DRE brain did not differ in terms of latency from the other classes. When looking at good outcomes only, HFO latency in the non-EpiR was longer than in the EpiR (*P* = 0.03), and similar to physiological HFOs (*P* > 0.05). The HFO amplitude variability across channels was lower for the physiological HFOs compared to HFOs from EpiR (0.2 versus 0.14, *P* < 0.001) or non-EpiR (0.21 versus 0.14, *P* < 0.001, Fig. 6D), but similar to the HFOs from the diffuse DRE brain, which also differed from the EpiR HFOs.

#### Spectral features

HFOs from diffuse DRE brain presented lower frequency (91 Hz) than physiological HFOs (96 Hz, *P* < 0.01) and non-EpiR HFOs (93 Hz, *P* < 0.01, Fig. 6E), while no differences were seen between the other classes. When including only patients with good outcome, the HFO frequency in the DRE brain (EpiR or non-EpiR) was lower than the physiological HFO frequency. Additionally, HFOs from both EpiR and non-EpiR exhibited higher power ratio (Fig. 6F) than physiological HFOs (*P* < 0.001) and diffuse DRE HFOs (*P* < 0.01; *P* = 0.02, Supplementary Table 2), and did not differ between each other.

#### Spatial features

The channel extent of EpiR HFOs (Fig. 6G) was larger than any other class of HFOs, i.e. physiological HFOs (*P* < 0.001), non-EpiR HFOs (*P* < 0.01) or HFOs from diffuse DRE brain. Channel extent of physiological HFOs was smaller than the diffuse DRE HFOs (24 versus 31, *P* < 0.001, Fig. 6G) but similar to the HFOs from the non-EpiR (24 versus 24, *P* = 0.51, Fig. 6G). When including good outcomes only, the source extent of HFOs from EpiR was smaller than physiological HFOs or HFOs from non-EpiR (2 versus 3, *P* < 0.001, 2 versus 3, *P* = 0.04 Fig. 6H), while the latter did not differ.

### MEG HFOs

We found a total of 39 physiological HFOs from TDC with MEG; for the DRE children, we found 11 HFOs from EpiR, 23 from non-EpiR and 69 from the diffuse DRE group.

#### Temporal and morphological features

Physiological HFOs showed a shorter propagation latency across channels than diffuse DRE HFOs (10 versus 16 ms, *P* = 0.005, Supplementary Table 3), without differences between the other HFO classes. HFO duration and its inter-channel variability did not differ between classes (Supplementary Table 3); yet, when we only included good outcome DRE patients, duration of physiological HFOs (79 ms) was shorter than HFOs from EpiR (148 ms, *P* = 0.004) or non-EpiR (116 ms, *P* = 0.003, Supplementary Table 3), and their duration variability was lower than in the non-EpiR (*P* = 0.03).

#### Spectral features

HFO frequency and power did not differ between classes. When including only good outcome DRE patients, we observed lower HFO frequency in EpiR than in physiological HFOs (94 versus 111 Hz, *P* = 0.014, Supplementary Table 3).

#### Spatial features

Physiological HFOs showed greater channel extent (17 versus 12, *P* = 0.009, and source extent (8 versus 3, *P* = 0.0007, Supplementary Table 3) than diffuse DRE HFOs.

### HFO classification performance

**Figure 7:**
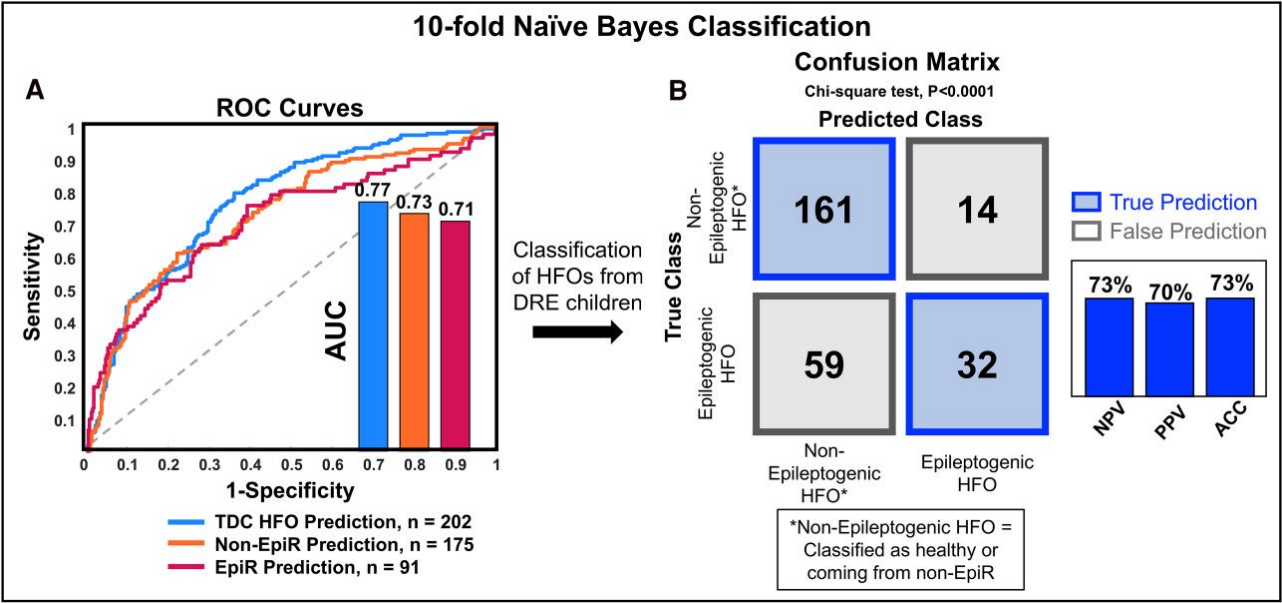
**Naïve-Bayes classification of HFOs recorded with HD-EEG (10-fold cross-validation)**. (**A**) ROC curves (one-versus-all binary classification) for each class of HFO (blue: TDC HFO versus others; orange: non-EpiR HFO from DRE versus others; red: EpiR HFO from DRE versus others). (**B**) Confusion matrix obtained for the classification of the HFOs recorded from the DRE group (*N* = 266) into epileptogenic versus non-epileptogenic. ACC, accuracy; AUC, area under the curve; DRE, drug-resistant epilepsy; EpiR, epileptogenic region; HFO, high frequency oscillation; non-EpiR, non-epileptogenic region; NPV, negative predictive value; PPV, positive predictive value; ROC, receiver operating characteristics; TDC, typically developing controls.

We trained the classifier on a dataset of 468 HFOs recorded with HD-EEG: 202 from healthy brain (class 1), 175 from non-EpiR (class 2) and 91 from EpiR (class 3). When looking at the one-versus-all binary classification for each class of HFO (Fig. 7A), we obtained an AUC of 0.77 for class 1 (physiological HFOs), 0.71 for class 2 (HFOs from non-EpiR) and 0.70 for class 3 (HFOs from EpiR). In terms of accuracy to classify HFOs from the DRE brain as either epileptogenic or not (Fig. 7B), we obtained an accuracy of 73%, with a negative and positive predictive value of 73% and 70% (*P* < 0.0001). The small size of the MEG HFO dataset did not allow us to test a classification algorithm: we had a total of 73 HFOs from MEG, where 39 were from healthy brain (class 1), 23 from Non-EpiR (class 2) and 11 from EpiR (class 3).

## Discussion

We present the first evidence that HFOs can be recorded and localized non-invasively from the healthy brain of TDC using HD-EEG and MEG; these purely physiological HFOs show distinctive features in terms of timing, morphology, spectrum and spatial distribution compared to HFOs recorded from children with DRE. We also present a unique cortical distribution map of physiological HFOs recorded from TDC using HD-EEG data co-registered on individual MRIs. Our main findings can be summarized as following: (i) HD-EEG proved superior to MEG in detecting HFOs from both the DRE and TDC group; (ii) physiological HFOs from TDC can be recorded and localized non-invasively, especially using HD-EEG, with maximal rates in central cortical regions; (iii) physiological HFOs from TDC present distinguishable temporal/morphological, spectral and spatial characteristics compared to HFOs from the DRE brain; and (iv) individual HFOs recorded from children with DRE can be classified as epileptogenic or not (through a multi-feature classification model) with an accuracy of 73%. Our findings mark a significant step forward in establishing non-invasive methods for using HFOs as epilepsy biomarkers and addressing critical gaps in the current literature on how to pinpoint pathological HFOs on non-invasive recordings.

### HFO rates in HD-EEG and MEG

We show that ripples can be recorded using HD-EEG or MEG in both DRE and TDC, with much higher rates in HD-EEG. Our data confirm findings from previous DRE studies^60^ demonstrating the superiority of EEG in detecting physiological HFOs.^35,36,58^ The lower occurrence of MEG ripples may be due to the distinct noise and sensitivity properties between the two modalities. While EEG is more susceptible to muscle artefacts, MEG is more affected by the environmental background noise.^61^ Moreover, the distance between HFO generators and MEG sensors leads to signal attenuation affecting the MEG detection rate; additionally, volume conduction further decreases the detectability of HFOs at the scalp level.^62-64^

### Correlation of HFO rates with age

In the DRE group, HD-EEG HFO rates were inversely correlated with patients’ age: the younger the child, the more the HFOs. Contrarily in TDC, no correlation was found, suggesting that the presence of physiological HFOs does not depend on the child's age while that of pathological HFOs does. This may indicate that the augmented HFO content in the younger children with DRE is linked to underlying epileptogenicity rather than physiological mechanisms. Differently, a prior study reported an inverse, nonlinear correlation between physiological HFO rate and participant age, which yet was spanning from infancy to adulthood.^24^ These different findings suggest that age-related changes in physiological HFOs may not become apparent across a longer age span. Our finding of reduced HFO rates with age in children with DRE aligns with previous observations.^65,66^ This result has significant implications for HFO detection on the scalp. As individuals age, both HFOs and spikes, which are the standard non-invasive EEG biomarkers, become less common, leading to a reduction in sensitivity over time. Interestingly, we did not find a difference in HFO rate between DRE patients with focal versus diffuse or generalized localization of epilepsy. This extends previous observations from other groups^65,67^ investigating HFOs in children and adolescents with different aetiologies using scalp EEG, stressing that our findings may be relevant to a broader population of children with DRE, not just those with drug-resistant focal seizures.

### Distribution of physiological HFOs

This study presents the first cortical map of ‘purely’ physiological HFOs (Fig. 5A) recorded from the paediatric healthy brain (TDC) using HD-EEG and ESI. Although we also recorded and localized physiological HFOs using MEG and MSI (Figs 3A and 4), the events were sporadic (median rates in all lobes, but the temporal, were null), thus their anatomical distribution across the cortex could not be further investigated.

As for the HD-EEG HFOs, we observed the highest rates of physiological HFOs in the sensorimotor central area (pre-and post-central gyrus) with a mean rate of 0.17 HFOs/min, followed by parietal, temporal and frontal regions with a similar mean rate between 0.09 and 0.12 HFOs/min. The strong presence of physiological HFOs in central areas is in line with prior iEEG studies where presumed physiological HFOs were often seen in the pre- and post-central regions.^10,13,18^ Our localization results also align with prior animal studies reporting spontaneous ripple activity in the sensory and association cortices during slow-wave sleep.^68^

Differently from prior iEEG studies,^9,10,20,24^ our data showed low rates of physiological HFOs in the occipital lobe (Fig. 5A), where ripples are known to be induced by visual stimuli but also spontaneously occurring during slow-wave sleep.^14^ An intracranial study demonstrated an increased rate of physiological HFOs in the occipital regions proximal to the calcarine cortex that was evident during adolescence and adulthood but not during infancy.^24^ In our study, however, the low rates of HFOs in the occipital lobe may be due the increased noise levels in the occipital EEG channels, as participants were sleeping on their back, reducing our ability to differentiate physiological HFOs from the noisy background interference. Furthermore, we should acknowledge the substantially different characteristics of our cohort (consisting of healthy children without any history of seizures) compared to the DRE cohorts investigated in prior iEEG works.

There is no prior report of physiological HFO localization in healthy TDC. Only a few EEG studies looked at physiological scalp HFOs from subjects without epilepsy,^37,66,69,70^ often reporting high rates of ripples in the central EEG channels^66^ (which aligns with our data), though without any localization information at the cortical level.

### Differences between physiological and pathological HFOs

We studied a comprehensive set of HFO features and compared them between physiological HFOs from TDC and different classes of HFOs from DRE (epileptogenic, non-epileptogenic or from diffusely epileptogenic brain). We found that purely physiological HFOs, compared to HFOs from the DRE brain, are shorter, have lower power and are more regular across EEG channels (both in time and amplitude); they also present with a longer latency between channels, suggesting a slower and shorter underlying propagation. Our findings also show that, in terms of spatial extent, physiological TDC HFOs differ from the epileptogenic HFOs in the focal DRE cases, but not from the non-epileptogenic HFOs. This observation indicates how these aspects may be key to pinpoint pathological HFOs within the DRE brain: epileptogenic HFOs present smaller source generators despite being seen in a larger number of channels. This may suggest a higher signal-to-noise ratio of these HFOs, which makes them ‘visible’ to more EEG channels, despite having a focal generator. Interestingly, when we quantified how many of the HFOs in the DRE group were temporally coupled with interictal spikes, as in our previous work,^71^ we observed that only 39% of them were coupled, with a similar proportion between epileptogenic and non-epileptogenic HFOs (41% and 39%). This suggests that the feature differences we observed are not necessarily due to the co-occurrence with a spike. Similarly, when looking at patients with good postsurgical outcome (where distinction between EpiR and non-EpiR is free of ambiguities), other temporal characteristics emerge to be potentially specific to the pathological HFOs (i.e. long duration and short latency).

#### Comparison with prior studies on HFO characteristics

Our findings represent a unique addition to the existing literature on the comparison between physiological and pathological events, given the non-invasive nature of our data, the comprehensiveness of the feature set and our cohort characteristics. Most prior studies compared a variety of characteristics between pathological and physiological HFOs using invasive EEG data or animal data.^9,21,72-75^ Similarly to our findings, a shorter duration and higher frequency of the physiological HFOs were reported invasively^9,21,72^ when comparing pathological HFOs recorded from the seizure onset zone (SOZ) and physiological HFOs (induced by visual/motor or recorded from outside the SOZ). Additionally, our data indicate that physiological HFOs are characterized by a more homogeneous (less variable) profile across channels. This is consistent with the findings from a prior study on single unit recording from rats^73^ with chronic temporal lobe epilepsy (versus controls), where control animals showed a consistent ripple amplitude throughout the recording session as opposed to animals with chronic epilepsy, where the ripple amplitude varied significantly. The regularity of the HFO amplitude profile was also studied in another iEEG study^74^ where HFOs presented lower amplitude variability (were more regular) outside than inside the SOZ. Our findings add to these previous studies by indicating that physiological HFOs also show a less variable duration than pathological ones.

We also showed that HFOs from EpiR have shorter latency compared to HFOs from non-EpiR or from the healthy brain. Several studies have explored the spatiotemporal characteristics of propagating interictal biomarkers of epilepsy,^36,39,40,76-79^ yet without any prior comparisons between HFOs from DRE patients and healthy participants. A previous study from our group^39^ found that HFO latencies in the resected tissue of patients with good outcomes were shorter than in poor outcomes. This finding suggests that short HFO latency may serve as a potential indicator of epileptogenicity as also indicated by our current findings. We also observed that pathological HFOs had a higher HFO-to-background power ratio compared to physiological HFOs. Similar findings were reported by another group^75^ investigating high-frequency background EEG activity in a cohort of patients with DRE using iEEG. They demonstrated that HFOs emerging from a quiet background (akin to our HFOs with a high-power ratio) were strongly associated with the SOZ, whereas HFOs arising from a continuous oscillatory background (akin to our HFOs with a low-power ratio) showed no such association. HFOs from a continuous oscillating background suggest non-specific and possibly physiological high-frequency activity in contrast to HFOs arising from a ‘flat’ background.

Finally, we demonstrated that EpiR HFOs had the fewest active cortical vertices. These results align with those reported by another group^73^ that showed that in animals with chronic epilepsy, a significantly smaller proportion of total CA1 neurons participated in ripple-like events compared to the proportion that were ripple-modulated in control animals, while an even sparser activation of neurons was observed during pathological HFOs.

### Classification of individual HFOs: epileptogenic or not?

Prior invasive studies^9,14,18,21,27,28,80,81^ have attempted to distinguish pathological (recorded from the SOZ, resected region, or associated with IEDs) from physiological HFOs (recorded from outside the SOZ, resected region, or not associated with IEDs) reaching moderately accuracies, yet without a definite group distinction. These prior studies were invasive and therefore suffered from the inability to validate their models using truly physiological HFOs. Here, we trained a classification algorithm to identify non-epileptogenic HFOs by means of not only data recorded from outside the EZ of patients with DRE but also using physiological HFOs from the brain of TDC. We then tested how well this could perform in the application scenario of interest, i.e. classifying the HFOs from DRE children as being epileptogenic or not, and obtained an overall validation accuracy of 73% (Fig. 7A). Most of the HFOs from the non-EpiR were correctly classified as non-epileptogenic (92% specificity; *N* = 161), with 37% classified as class 1 and 63% as class 2. This suggests that, while some HFOs from non-EpiR resemble truly physiological HFOs from TDC (class 1), others do not but show a feature profile that is distinguishable from the epileptogenic HFOs (as we could also see via the individual feature analysis discussed above, Fig. 6A–H). Interestingly, 45% of the HFO recorded from the EpiR were classified as such (low sensitivity), which highlights the need for a larger and more robust validation sample (exclusively consisting of seizure-free surgical cases) to accurately assess performance. Moreover, while the overall validation accuracy of 73% is encouraging, particularly in distinguishing non-epileptogenic from epileptogenic HFOs, clinical applications may demand higher performance (e.g. >90%).^82,83^ A previous intracranial study demonstrated the link between damage to brain areas exhibiting physiological HFOs and the postsurgical occurrence of speech impairments.^31^ This finding emphasizes the need for a careful and accurate differentiation between epileptogenic and non-epileptogenic HFOs, which are not associated with seizures. Understanding this distinction is essential for clinicians when making informed decisions regarding treatment options. Proper identification can help optimize surgical outcomes minimizing postoperative complications, particularly in eloquent areas of the brain. Therefore, while 73% accuracy is an encouraging benchmark, ongoing development and optimization are crucial for direct implementation in clinical practice, where greater sensitivity and wider validation are needed. We warrant larger multi-centric scalp EEG studies to further validate the HFO classification model and feature set we present here.

## Conclusions

We provide first evidence that physiological HFOs can be detected and localized non-invasively in TDC via HD-EEG and MEG. In our data, physiological HFOs from TDC localize mostly in central cortical regions and exhibit distinct temporal, spectral and spatial characteristics when compared to those generated by the epileptic brain. Using truly physiological HFOs for training, we were able to classify individual HFOs recorded from children with DRE as coming from epileptogenic or non-epileptogenic regions with high specificity. Our study highlights the importance of non-invasive, whole-head recordings from healthy controls in differentiating physiological from pathological HFOs. This approach lays the foundation for developing non-invasive high-frequency biomarkers to delineate the EZ. Such advances are crucial for enhancing the clinical management of paediatric epilepsy, as they could improve presurgical evaluations and outcomes, while reducing reliance on invasive procedures and surgical risks.

## Supplementary Material

fcaf170_Supplementary_Data

## Data Availability

Data can be made available after request to the corresponding author. The codes used in this study are available at: https://github.com/LorenzoFabbri95/Noninvasive-Classification-of-Physiological-and-Pathological-High-Frequency-Oscillations-in-Children.git.
