# Supplementary material for: Noninvasive classification of physiological and pathological high frequency oscillations in children
Source: Brain Commun. 2025 May 2;7(3):fcaf170. doi: 10.1093/braincomms/fcaf170 (PMC12077393; doi:10.1093/braincomms/fcaf170)
Supplement: fcaf170_Supplementary_Data [file fcaf170_supplementary_data.docx]

| **Supplementary Table 1. Demographic and Clinical Characteristics of DRE Cohort** | | | | | | | | | | | |
| --- | --- | --- | --- | --- | --- | --- | --- | --- | --- | --- | --- |
| **Pt** | **Sex** | **Age [y]** | **Age Onset [y]** | **Preoperative MRI Findings** | **Side** | **Epileptogenic Regions*** | **sEEG Coverage** (N. Electrodes, N. Contacts) | **Type of Surgery** | **Outcome (f/u)** | **Site** | **Modality** |
| 1 | M | 16 | 4 | L Hip | GEN | N/A | N/A | N/A | N/A | CCH | MEG |
| 2 | F | 17 | 1 | FCD (R F) | R | F (MRI, Surgery) | T(5), F(2), Cing (2)  (9, 116) | Ablation (R Ant Ins) | 1 | CCH | EEG |
| 3 | M | 9 | 1.5 | Normal | GEN | N/A | N/A | N/A | N/A | CCH | EEG |
| 4 | F | 12 | 4 | L F FCD | L | F (MRI, Surgery) | T(2), Cing (3), F/C (4)  (9, 134) | Ablation (LF) | 1 (24 mo) | CCH | EEG |
| 5 | F | 7 | 6 | Subtle/Normal | GEN | N/A | N/A | N/A | N/A | CCH | EEG |
| 6 | F | 9 | 1.75 | Normal | GEN | N/A | N/A | N/A | N/A | CCH | EEG |
| 7 | F | 6 | 3 | Tumor  (L mes T) | L | T (MRI, ictal EEG, Surgery) | N/A | One-stage Resection LT | 1 (6 mo) | CCH | EEG+MEG |
| 8 | F | 15 | 7 | L Hem injury (T, Ins, Hip) | L | FCPT (MRI, ictal EEG) | N/A | N/A | N/A | CCH | EEG |
| 9 | M | 14 | 13 | Bil Hip Edema | LR | Bil T (MRI, SEEG) | RT(11), RP(1) (12,154) | Ablation (L Hip/Amyg) | 4 (6mo) | CCH | EEG+MEG |
| 10 | M | 15 | 2 | Encephalomalacia and FCD1B s/p L ATL | LR | T (ictal EEG) | RT(6), LT(6) LO(1), LP(1)  (14, 140) | RNS (R T/Hip) | 4 (24 mo) | CCH | EEG+MEG |
| 11 | F | 9 | 0.08 | R FP Parasag/Cing | R | FPC Cing (MRI) | N/A | One-stage RF Resection | 4 (6 mo) | CCH | EEG+MEG |
| 12 | M | 12 | 11 | FCD (RF) | R | F (MRI, ictal EEG) | N/A | One-stage RF Resection | 1 (6 mo) | CCH | EEG+MEG |
| 13 | F | 17 | 6 | L MTS | L | T (MRI, Surgery) | T(6), P(2), C(1)  (9, 112) | Amyg-Hip | 1 (12 mo) | CCH | MEG |
| 14 | M | 17 | 16 | L Amyg (suspicious FCD) | L | T (MRI, ictal EEG/SEEG) | T (8, 98) | LT Resection | 3 (12 mo) | CCH | EEG+MEG |
| 15 | F | 8 | 7 | Lesion (LT) | L | T (MRI, ictal EEG) | N/A | LT lobectomy | 1 (24 mo) | CCH | EEG+MEG |
| 16 | F | 15 | 3 | Hippocampal sclerosis (R) | R | T (MRI, ictal EEG) | N/A | N/A | N/A | CCH | EEG+MEG |
| 17 | M | 9 | 5 | Mild volume loss | GEN | N/A | LT(2), L Cing (2), R Cing (3), R T(3) R F (4)  (14, 174) | N/A | N/A | CCH | EEG+MEG |
| 18 | F | 10 | 3 | FCD (LP, LT, R PO) | GEN | N/A | R Cing (2), RF(1), R P/O (1), RT (3), LT(4), LF (1), L Cing(2), LP(1)  (15,194) | N/A | N/A | CCH | EEG+MEG |
| 19 | F | 12 | 9 | Hemangiomas (R) | L | TC (ictal EEG) | RT(5), LT(5)  (10, 114) | Ablation (LT) | 4 (6 mo) | CCH | EEG+MEG |
| 20 | F | 14 | 6 | Normal | L | FT (ictal EEG) | N/A | N/A | N/A | CCH | MEG |
| 21 | F | 11 | 6 | Ischemic Injury (R basal ganglia) | R | FTC (ictal EEG, SEEG) | T(6), C(2), F(2) (10,82) | RNS (RC) | no f/u | CCH | EEG+MEG |
| 22 | M | 17 | 10 | Normal | GEN | N/A | N/A | N/A | N/A | CCH | EEG+MEG |
| 23 | M | 15 | 0.25 | Hippocampal sclerosis (L) | L | FT Cing (MRI + sEEG) | T(6), C(2), Cing(3), F(2)  (13, 134) | LF resection | 3 (12 mo) | CCH | EEG+MEG |
| 24 | F | 13 | 11 | FCD (R ant Cing) | GEN | N/A | N/A | N/A | N/A | CCH | EEG+MEG |
| 25 | M | 16 | 13 | Mild volume loss (supraentorial) | GEN | N/A | N/A | N/A | N/A | CCH | MEG |
| 26 | F | 14 | 3 | Volume loss (supratentorial, O, P) | R | TOP (SEEG) | T(4), O(2), P(3)  (9, 106) | N/A | N/A | CCH | EEG+MEG |
| 27 | F | 15 | 9 | Normal | L | LTP (SEEG) | Cing(6), F(2), T(1) | RNS L Ins and L P | no f/u | CCH | EEG+MEG |
| 28 | F | 12 | 11 | FCD (RT) | R | T (MRI, EEG) | N/A | N/A | N/A | CCH | EEG+MEG |
| 29 | F | 16 | 0.25 | Hippocampal Sclerosis (L) | L | T (MRI, Surgery) | N/A | Ablation (LT) | 1 | CCH | EEG+MEG |
| 30 | M | 17 | 0 | Encephalomalacia (L MCA) | L | FC (MRI, Surgery) | N/A | L FC resection | 1 | CCH | MEG |
| 31 | F | 13 | 0.08 | Hippocampal Sclerosis (LR), Encephalomalacia (LT), Cavernomas | GEN | N/A | N/A | N/A | N/A | CCH | MEG |
| 32 | F | 14 | 1.5 | Normal | GEN | N/A | N/A | N/A | N/A | CCH | EEG+MEG |
| 33 | F | 8 | 2.5 | FCD (L Ins, LPO) | L | TOP  (MRI, ictal EEG) | F(3),P(1), P(3), T(2), L(1)  (9,198) | N/A | N/A | CCH | EEG+MEG |
| 34 | F | 15 | 13 | Chiari I Malformation | GEN | N/A | N/A | N/A | N/A | CCH | EEG+MEG |
| 35 | F | 15 | 14 | Normal | GEN | N/A | N/A | N/A | N/A | CCH | EEG+MEG |
| 36 | M | 15 | 11 | FCD (RF) | R | F | T(3), F(3), Cing (3), P (1)  (10, 120) | RF resection | 1  (3 mo) | CCH | EEG+MEG |
| 37 | F | 17 | 7 | Normal | R | P | T(7), Cing (2), P(3) | N/A | N/A | CCH | EEG+MEG |
| 38 | M | 18 | 6 | Postoperative changes (L Hip) s/p ablation | L | T | T(7, 64) | Ablation (LT) | N/A | CCH | MEG |
| 39 | M | 5 | 2 | FCD (LF) | L | FC (MRI + ictal SOZ) | N/A | N/A | N/A | CCH | EEG |
| 40 | M | 9 | 4 | s/p Ablation (RF) | R | FC | N/A | N/A | N/A | CCH | EEG+MEG |
| 41 | M | 17 | 16 | Normal | L | T (ictal EEG) | N/A | N/A | N/A | CCH | EEG+MEG |
| 42 | F | 13 | 7.5 | Normal | L | FC (ictal EEG) | N/A | N/A | N/A | CCH | EEG+MEG |
| 43 | F | 7 | 0.5 | Non-specific | GEN | GEN | N/A | N/A | N/A | CCH | EEG+MEG |
| 44 | F | 17 | 2.5 | s/p LF resection | L | F (ictal EEG) | N/A | RNS (LF) | N/A | CCH | EEG+MEG |
| 45 | M | 10 | 9.5 | FCD (RF), s/p LITT (R subependymal heterotopia) | R | P F Cing  (MRI + SEEG/Surgery) | F(7), C(7), P(7), T(5)  (26, 253) | Ablation (R P,F,Cing) | 3 (6 mo) | BCH | EEG+MEG |
| 46 | M | 15 | 10.5 | Encephalomalacia (LP) | L | TP (MRI, SEEG) | Post F(2), C(4), T(4), P(3), O(2)  (15, 160) | Ablation (LT) | 3 (24 mo) | BCH | EEG+MEG |
| 47 | M | 11 | 3 | Sclerosis (LT), gyriform calcifications (L FP) | L | TP (MRI + SEEG/Surgery) | T(7), P(4), O(1)  (12,120) | Ablation (L T,P) | 1 (6 mo) | BCH | EEG+MEG |
| 48 | F | 15 | 12 | FCD (LT) | L | T (MRI, SEEG/Surgery) | T(8), C(1), F(2), O (1)  (12, 138) | Resection (LT) | 1 (6 mo) | BCH | EEG+MEG |
| 49 | M | 10 | 8 | FCD (LT) | L | T (MRI, SEEG/Surgery) | T(9), F(2), C(1), P(2), O(1)  (14, 164) | Resection (LT) | 1 (18 mo) | BCH | EEG+MEG |
| 50 | M | 14 | 10 | Encephalomalacia (R>L) | GEN | N/A | N/A | N/A | N/A | BCH | EEG+MEG |
| 51 | F | 8 | 8 | Normal | L | PC (SEEG) | F(4), C(6), P(3)  (13,128) | Ablation (L P,C) | 3/4 (18 mo) | BCH | EEG+MEG |
| 52 | F | 11 | 8 | Normal | L | F (SEEG/Surgery) | L F(6), C(7), P(6) | Ablation (LF) | 2  (24 mo) | BCH | EEG+MEG |
| 53 | F | 14 | 11 | FCD (LT) | L | T (MRI, SEEG/Surgery) | N/A | Ablation (LT) | 1 (12 mo) | BCH | EEG+MEG |
| 54 | F | 10 | 6 | FCD (F) | R | FT (MRI, SEEG/Surgery) | N/A | 1st: Ablation (R Ins)  2nd: Ablation (R F) | 1st: 3-4  (6 mo)  2nd: no f/u | BCH | EEG+MEG |

F = Female; M = Male; Age = age at epilepsy surgery; L = Left; R = Right; T= Temporal; F = Frontal; P = Parietal; C = Central; O = Occipital; Gen = generalized; Ins = Insula; Hip = Hippocampus; Cing = cingulate; Amyg = amygdala; Hem = hemisphere; Ant = anterior; Post = posterior; Bil = bilateral, Parasag = parasagittal; FCD = focal cortical dysplasia; MTS = mesial temporal sclerosisy = years; ATL = anterior temporal lobectomy; MCA = middle cerebral artery; mo = months; f/u = follow-up; s/p = status post; SEEG = stereoelectroencephalography; SOZ = seizure onset zone; RNS = responsive neurostimulation; LITT = laser interstitial thermal therapy; CCH = cook children’s hospital; BCH = boston children hospital

| **Supplementary Table 2. Kruskal-Wallis test on HFO features** | | | | | | | | | | | | |
| --- | --- | --- | --- | --- | --- | --- | --- | --- | --- | --- | --- | --- |
| **Feature** | **Patients** | **TDC**  **Median (IQR)** | **Non-EpiR**  **Median (IQR)** | **EpiR**  **Median (IQR)** | **Gen**  **Median (IQR)** | ***P*-value (Kruskal-Wallis)** | | | | | | |
|  |  |  |  |  |  | All groups | TDC vs Non-EpiR | TDC vs EpiR | TDC vs Gen | EpiR vs Non-EpiR | EpiR vs Gen | Non-EpiR vs Gen |
| **HD-EEG** | | | | | | | | | | | | |
| **Duration [ms]** | All | 81  (60-100) | 103  (76-145) | 118  (82-182) | 100  (71-150) | **<0.0001** | **<0.0001 [-0.49]** | **<0.0001 [-0.8]** | **<0.0001 [-0.54]** | 0.16 | ~1 | 0.20 |
|  | G.O. | - | 108  (76-163) | 163  (92-216) | *-* | **<0.0001** | **<0.0001 [-0.69]** | **<0.0001 [-1.19]** | *-* | 0.08 | *-* | *-* |
| **Duration Variability** | All | 0.18  (0.11-0.25) | 0.22  (0.15-0.31) | 0.25  (0.17-0.34) | 0.23  (0.14-0.30) | **<0.0001** | **0.003 [-0.23]** | **0.0001 [-0.37]** | **0.01 [-0.17]** | 0.48 | ~1 | 0.59 |
|  | G.O. | - | 0.22  (0.12-0.31) | 0.26  (0.14-0.35) | *-* | **0.003** | 0.18 | **0.003 [-0.3]** | *-* | 0.32 | *-* | *-* |
| **Propagation Latency [ms]** | All | 9.3  (5.2-18.3) | 7.4  (3.3-14.2) | 5.9  (3.6-11.3) | 6.4  (4.2-13.1) | **0.003** | 0.12 | **0.003 [0.19]** | 0.16 | 0.35 | ~1 | 0.51 |
|  | G.O. | - | 9.3  (5.5-23.3) | 6.2  (4.4-12) | *-* | **0.03** | 0.7 | 0.07 | *-* | **0.03 [-0.5]** | *-* | *-* |
| **Amplitude Variability** | All | 0.14  (0.09-0.19) | 0.21  (0.14-0.28) | 0.20  (0.14-0.28) | 0.16  (0.12-0.24) | **<0.0001** | **<0.0001 [-0.58]** | **<0.0001 [-0.58]** | 0.1 | 0.98 | **0.008 [0.37]** | 0.07 |
|  | G.O. | - | 0.22  (0.11-0.28) | 0.19  (0.13-0.28) | *-* | **<0.0001** | **0.001 [-0.43]** | **0.004 [-0.48]** | *-* | ~1 | *-* | *-* |
| **Frequency [Hz]** | All | 96  (90-103) | 93  (88-101) | 96  (91-107) | 91.4  (84.5-101.5) | **0.0007** | 0.09 | ~1 | **0.002 [0.31]** | 0.18 | 0.41 | **0.008 [0.12]** |
|  | G.O. | - | 91  (87-98) | 92  (89-98) | *-* | **0.001** | **0.004 [0.37]** | **0.04 [0.39]** | *-* | 0.93 | *-* | *-* |
| **Power Ratio** | All | 0.60  (0.58-0.61) | 0.62  (0.59-0.64) | 0.61  (0.59-0.66) | 0.60  (0.57-0.63) | **<0.0001** | **<0.0001 [-0.38]** | **0.0001 [-0.46]** | 0.73 | ~1 | **0.007 [0.36]** | **0.02 [0.29]** |
|  | G.O. | - | 0.61  (0.59-0.64) | 0.61  (0.58-0.68) | *-* | **0.0001** | **0.003 [-0.15]** | **0.002 [-0.47]** | *-* | 0.89 | *-* | *-* |
| **Channel Extent** | All | 24  (20-30) | 24  (18-46) | 31  (23-45) | 31  (23-41) | **<0.0001** | 0.51 | **<0.0001 [-0.8]** | **<0.0001 [-0.72]** | **0.006 [0.14]** | **0.008 [0.14]** | ~1 |
|  | G.O. | - | 22  (17-25) | 30  (21-38) | *-* | **<0.0001** | **0.01 [0.18]** | **0.04 [-0.57]** | *-* | **<0.0001 [0.56]** | - | - |
| **Source Extent** | All | 3  (2-6) | 3  (2-6) | 2  (1-4) | 5  (3-9) | **<0.0001** | ~1 | 0.07 | **0.002 [-0.11]** | 0.1 | **0.002 [-0.45]** | **<0.0001 [-0.62]** |
|  | G.O. | - | 3  (2-5) | 2  (1-3) | *-* | **<0.0001** | 0.53 | **0.0004 [0.41]** | *-* | **0.04 [-0.36]** | - | - |

TDC = typically developing children; IQR = interquartile range; Non-EpiR = non-epileptogenic regions; EpiR = epileptogenic bc regions; Gen = generalized; G.O. = good outcome; [] = effect size (Cohen’s d)

| **Supplementary Table 3. Kruskal-Wallis test on HFO features** | | | | | | | | | | | | |
| --- | --- | --- | --- | --- | --- | --- | --- | --- | --- | --- | --- | --- |
| **Feature** | **Patients** | **TDC**  **Median (IQR)** | **Non-EpiR**  **Median (IQR)** | **EpiR**  **Median (IQR)** | **Gen**  **Median (IQR)** | ***P*-value (Kruskal-Wallis)** | | | | | | |
|  |  |  |  |  |  | All groups | TDC vs Non-EpiR | TDC vs EpiR | TDC vs Gen | EpiR vs Non-EpiR | EpiR vs Gen | Non-EpiR vs Gen |
| **MEG** | | | | | | | | | | | | |
| **Duration [ms]** | All | 79  (65-100) | 103  (77-129) | 105  (73-146) | 81  (67-96) | 0.08 | 0.11 | 0.32 | 0.86 | ~1 | 0.28 | 0.56 |
|  | G.O. | - | 116  (70-141) | 148  (129-223) | *-* | **0.0001** | **0.003 [-1.5]** | **0.004 [-2.4]** | *-* | 0.83 | *-* | *-* |
| **Duration Variability** | All | 0.19  (0.14-0.27) | 0.24  (0.14-0.4) | 0.3  (0.11-0.39) | 0.21  (0.15-0.27) | 0.31 | 0.47 | 0.38 | 0.8 | 0.97 | 0.85 | 0.69 |
|  | G.O. | - | 0.27  (0.14-0.35) | 0.34  (0.22-0.42) | *-* | **0.016** | **0.03 [-1.3]** | 0.24 | *-* | 0.93 | *-* | *-* |
| **Propagation Latency [ms]** | All | 9.8  (6-16) | 15.6  (10-19) | 15.6  (11-22) | 14.8  (11-21) | **0.007** | 0.16 | 0.15 | **0.005 [-0.71]** | 0.97 | 0.97 | ~1 |
|  | G.O. | - | 14.6  (9-21) | 19.3  (12-25) | *-* | 0.11 | 0.76 | 0.1 | *-* | 0.4 | *-* | *-* |
| **Amplitude Variability** | All | 0.17  (0.12-0.21) | 0.17  (0.12-0.24) | 0.18  (0.12-0.21) | 0.15  (0.09-0.21) | 0.67 | ~1 | ~1 | 0.7 | ~1 | 0.86 | 0.91 |
|  | G.O. | - | 0.17  (0.12-0.22) | 0.17  (0.13-0.2) | *-* | 0.96 | 0.98 | 0.95 | *-* | ~1 | *-* | *-* |
| **Frequency [Hz]** | All | 111  (104-122) | 114  (102-123) | 103  (95-119) | 116  (102-122) | 0.46 | ~1 | 0.57 | 0.98 | 0.5 | ~1 | 0.39 |
|  | G.O. | - | 111  (104-122) | 94  (91-131) | *-* | **0.01** | 0.41 | **0.014 [1.5]** | *-* | 0.27 | *-* | *-* |
| **Power Ratio** | All | 0.59  (0.57-0.61) | 0.58  (0.56-0.61) | 0.59  (0.58-0.61) | 0.58  (0.57-0.6) | 0.19 | 0.6 | ~1 | 0.19 | 0.84 | 0.99 | 0.65 |
|  | G.O. | - | 0.6  (0.58-0.61) | 0.61  (0.6-0.62) | *-* | **0.02** | 0.07 | 0.12 | *-* | 0.98 | *-* | *-* |
| **Channel Extent** | All | 17  (12-28) | 11  (10-20) | 16  (13-20) | 12  (9-16) | **0.006** | 0.14 | ~1 | **0.009 [0.5]** | 0.36 | ~1 | 0.18 |
|  | G.O. | - | 13  (10-24) | 20  (17-32) | *-* | 0.27 | 0.97 | 0.54 | *-* | 0.74 | *-* | *-* |
| **Source Extent** | All | 8  (4-11) | 5  (3-11) | 6  (3-10) | 3  (1-6) | **0.0006** | 0.96 | 0.85 | **0.0007 [0.56]** | 0.98 | **0.04 [0.3]** | 0.43 |
|  | G.O. | - | 8  (4-17) | 6  (5-12) | *-* | 0.99 | 0.99 | ~1 | *-* | ~1 | *-* | *-* |

TDC = typically developing children; IQR = interquartile range; Non-EpiR = non-epileptogenic regions; EpiR = epileptogenic bc regions; Gen = generalized; G.O. = good outcome; [] = effect size (Cohen’s d)

**Supplementary Table 4 | Median duration of NREM sleep analyzed**

| **Group** | **Median [min]** | **IQR [25%-75%]** |
| --- | --- | --- |
| EEG DRE | 5.7 | 3.8-7.8 |
| EEG TDC | 4.9 | 2.9-6.6 |
| MEG DRE | 7.8 | 6.3-8.5 |
| MEG TDC | 6.6 | 6.1-8.4 |

DRE = drug-resistant epilepsy; TDC = typically developing controls; IQR = interquartile range
